# False and partial Eisenstein-type series related to unimodal sequences

**DOI:** 10.1007/s40687-026-00609-y

**Published:** 2026-05-06

**Authors:** Kathrin Bringmann, Badri Vishal Pandey, Jan-Willem van Ittersum

**Affiliations:** 1 Department of Mathematics and Computer Science, University of Cologne, Weyertal 86-90, 50931 Cologne, Germany; 2 Present Address: Current address: Korteweg–de Vries Institute for Mathematics, University of Amsterdam, Postbus 94248, 1090 GE, Amsterdam, Netherlands

**Keywords:** Eisenstein series, False theta functions, Partial theta functions, Unimodal sequences, 11F03, 11F11, 11F37, 11F50, 11P82

## Abstract

Motivated by the fact that the classical Jacobi theta function $\vartheta$ is the exponential generating function of the Eisenstein series, we study the exponential Taylor coefficients (in the elliptic variable) of a related natural partial theta function, as well as a false theta function corresponding to the Dedekind eta function. We prove that the space spanned by these objects is closed under differentiation, analogous to the space of quasimodular forms, and that it contains the quasimodular forms themselves. We further provide their Fourier expansions, establish quasimodular completions, and derive a recursive formula for the Taylor coefficients of the logarithm of the unimodal rank generating function, expressed as partition traces of the false and partial objects.

## Introduction and statement of results

The *Jacobi theta function* ($\zeta :=e^{2\pi i z}, q:=e^{2\pi i\tau }, z\in \mathbb {C},\tau$ in the complex upper half plane $\mathbb H$)

1.1 $$\begin{aligned} \vartheta (z;\tau ):={i}\sum _{n\in \mathbb {Z}} (-1)^n q^{\frac{1}{2}\left( n+\frac{1}{2}\right) ^2}\zeta ^{n+\frac{1}{2}} = -iq^{\frac{1}{8}} \zeta ^{-\frac{1}{2}} (q)_\infty (\zeta )_\infty (\zeta ^{-1}q)_\infty , \end{aligned}$$

where $(a)_n:= \prod _{j=0}^{n-1} (1-aq^j)$ for $n\in \mathbb {N}_0\cup \{\infty \}$, and its generalizations serve as fundamental building blocks for elliptic functions and modular forms. Through the so-called theta decomposition they describe the structure of the space of Jacobi forms in terms of vector-valued modular forms. Furthermore, these functions appear in various areas, such as number theory, combinatorics, and mathematical physics, as well as partition theory and string theory (see [6, 8–10]).

A classical observation is that the Taylor expansion of $\vartheta$ involves Eisenstein series. More precisely, up to normalization, $\vartheta$ is the exponential generating function of the *Eisenstein series* $G_k$ [15, (7)], defined for weights $k\in \mathbb {N}$ by

1.2 $$\begin{aligned} G_{k}(\tau ):={\left\{ \begin{array}{ll}-\frac{B_k}{2k}+\sum _{n\ge 1} \sigma _{k-1}(n)q^n &  \text {if }2\mid k,\\ 0 &  \text {otherwise,}\end{array}\right. } \end{aligned}$$

where $\sigma _{k-1}$ denotes the *divisor function* $\sigma _{k-1}(n):=\sum _{d\mid n}d^{k-1}$. Indeed, we have

1.3 $$\begin{aligned} \vartheta (z;\tau )&=-2\pi z \eta ^3(\tau )\exp \left( - 2\sum _{k\ge 1} G_{k}(\tau )\frac{(2\pi iz)^k}{k!}\right) , \end{aligned}$$

where $\eta (\tau ):=q^{\frac{1}{24}}\prod _{n\ge 1}(1-q^n)$ denotes the *Dedekind eta function*. This naturally raises the question of whether similar exponential expansions exist for other theta-type functions. In this paper, we consider false and partial theta functions introduced by Rogers [13] and Ramanujan [11], respectively. Unlike $\vartheta$, these functions are no longer Jacobi forms, but they retain striking modular-like properties. To be precise, we define the partial and false theta functions *T* and *h* by^1^

1.4 $$\begin{aligned} T(z;\tau )&:=2i\sum _{n\ge 0} (-1)^n \zeta ^{n+\frac{1}{2}} q^{\frac{1}{2}\left( n+\frac{1}{2}\right) ^2},\end{aligned}$$

1.5 $$\begin{aligned} h(\zeta ;q)&:= (1 - \zeta ) \sum _{n\ge 0} (-1)^n \zeta ^{3n} q^{\frac{n(3n+1)}{2}} \left( 1 - \zeta ^2 q^{2n+1}\right) \nonumber \\&= \sum _{n\in \mathbb {Z}} (-1)^n \operatorname {sgn}\left( n+\frac{1}{2}\right) \left( \zeta ^{\operatorname {sgn}\left( n+\frac{1}{2}\right) 3n}-\zeta ^{\operatorname {sgn}\left( n+\frac{1}{2}\right) (3n+1)}\right) q^{\frac{n(3n+1)}{2}}. \end{aligned}$$

Our aim is to study Eisenstein-type properties for the exponential Taylor coefficients of *T* and *h*. That is, we investigate the sequences of functions $\{g_k\}_{k\in \mathbb {N}}$ and $\{h_k\}_{k\in \mathbb {N}}$, which we refer to as *partial* and *false Eisenstein-type series*, respectively, defined implicitly by

1.6 $$\begin{aligned}&T_0(\tau )\exp \left( - \sum _{k\ge 1}g_k(\tau )\frac{(2\pi iz)^k}{k!}\right) := {T(z;\tau )}, \qquad \left( T_0(\tau ):= T(0;\tau )\right) \end{aligned}$$

1.7 $$\begin{aligned}&\quad -2i\sin (\pi z)q^{-\frac{1}{24}}\eta (\tau )\exp \left( -\sum _{k\ge 1}h_k(\tau )\frac{(2\pi iz)^k}{k!}\right) := h(\zeta ;q). \end{aligned}$$

The functions $g_k$ and $h_k$ are neither modular nor quasimodular, and do they do not appear to admit quasimodular completions in the classical sense.^2^ Instead, they admit a modular completion depending on an additional independent variable $w\in \mathbb {H}$. More precisely, a function $\widehat{f}(\tau ,\overline{\tau },w,\overline{w})$ is a *(quasi)modular completion* of *f* if $\widehat{f}$ transforms (quasi)modularly under the simultaneous action $(\tau ,w)\mapsto (\frac{a\tau +b}{c\tau +d},\,\frac{aw+b}{cw+d})$ and such that the original function *f* is recovered as a limiting value of $\widehat{f}$. In particular, in our setting, we have, for any $\varepsilon >0$ (see Sect. 2.2),

$$\begin{aligned} f(\tau )=\lim _{t\rightarrow \infty }\widehat{f}(\tau ,\overline{\tau },\tau +it+\varepsilon ,\overline{\tau }-it+\varepsilon ). \end{aligned}$$

To state our result, for $\tau ,w\in \mathbb {H}$ with $\tau \ne w$ and , define

1.8 $$\begin{aligned} \chi _{\tau ,w} \left( \begin{matrix}a& b\\ c& d\end{matrix}\right) := \sqrt{\frac{i(w-\tau )}{(c\tau +d)(cw+d)}} \frac{\sqrt{c\tau +d}\sqrt{cw+d}}{\sqrt{i(w-\tau )}}\in \{1,-1\}. \end{aligned}$$

We let $\delta _{j,\ell }$ denote the Kronecker delta, i.e., $\delta _{j,\ell }=1$ if $j=\ell$ and $\delta _{j,\ell }=0$ otherwise.

### Theorem 1.1

There exist functions $\widehat{g}_k(\tau ,w)$ such that the following hold:

1. For , we have
  $$\begin{aligned} \widehat{g}_{k} \left( \frac{a\tau +b}{c\tau +d},\frac{aw+b}{cw+d}\right)&= \chi ^{k}_{\tau ,w} (\gamma ) (c\tau +d)^k \widehat{g}_{k}(\tau ,w) + \delta _{k,2}\frac{ic}{2\pi }(c\tau +d) . \end{aligned}$$
2. For any $\varepsilon >0$, we have
  $$\begin{aligned} \lim _{t\rightarrow \infty } \widehat{g}_{k}(\tau ,\tau +it+\varepsilon ) = g_k(\tau ) . \end{aligned}$$

For $h_k$, we have the following result on their completions.

### Theorem 1.2

There exist functions $\widehat{h}_k(\tau ,w)$ such that the following hold:

1. For , we have
  $$\begin{aligned} \widehat{h}_{k} \left( \frac{a\tau +b}{c\tau +d},\frac{aw+b}{cw+d}\right)&= \chi ^{k}_{\tau ,w} (\gamma ) (c\tau +d)^k \widehat{h}_{k}(\tau ,w) +\delta _{k,2} \frac{3 i c}{2 \pi }(c\tau +d). \end{aligned}$$
2. For any $\varepsilon >0$, we have
  $$\begin{aligned} \lim _{t\rightarrow \infty } \widehat{h}_{k}(\tau ,\tau +it+\varepsilon ) =h_k(\tau ). \end{aligned}$$

A classical fact is that the algebra of Eisenstein series is closed under differentiation (see (2.4)). Recently, the authors introduced a new class of Eisenstein-type series arising from the so-called rank generating function and proved that these objects are likewise closed under differentiation (see [4, Theorem 1.2 (3)]). Rausch [12] then constructed a family of Eisenstein-type series associated with so-called *k*-ranks, which also satisfy a closedness property. In this paper, we show that the algebra generated by the functions $g_k$ (and $h_k$) admits the same type of differential structure. More precisely, let $\widetilde{\mathcal {M}}:=\mathbb {C}[G_2,G_4,G_6]$ be the space of quasimodular forms,

$$ \mathcal {T}:=\mathbb {C} \left[ g_1,g_2,g_3,\ldots \right] , \quad \mathcal {H}:=\mathbb {C} \left[ h_1,h_2,h_3\ldots \right] , \quad \text {and} \quad D:=q\frac{\partial }{\partial q}. $$

### Theorem 1.3

The space $\mathcal {T}$ is closed under the action of *D*. That is, for $k\in \mathbb {N}$, we have

$$\begin{aligned} D(g_k) = \frac{g_{k+2}}{2} - \frac{1}{2}\sum _{d=0}^{k} \left( {\begin{smallmatrix}k\\ \\ d\end{smallmatrix}}\right) g_{d+1} g_{k-d+1},\qquad D(\textrm{Log}\left( T_0\right) ) = -\frac{g_2}{2} +\frac{g_1^2}{2}. \end{aligned}$$

Furthermore the space $\mathcal {H}$ is a differential algebra extending the space of quasimodular forms:

### Theorem 1.4

The space $\mathcal {H}$ is closed under the action of *D*. More precisely, for $k\in \mathbb {N}$, we have

$$\begin{aligned} D(h_k)&= \frac{h_{k+2}}{6} -\frac{1}{6}\sum _{d=0}^k \left( {\begin{smallmatrix}k\\ \\ d\end{smallmatrix}}\right) h_{d+1}h_{k-d+1},\quad \frac{h_2}{6} - \frac{h_1^2}{6} = G_2. \end{aligned}$$

Further, we have $\mathcal {H} =\widetilde{\mathcal {M}}[h_1,h_3,h_5,\ldots ]$.

**Remarks 1 **

1. By Theorem 1.4, the space of quasimodular forms for $\textrm{SL}_2(\mathbb {Z})$ is contained in $\mathcal {H}$, even though the completions of their generators $h_k$ have quasi completions which transform on $\Gamma _0(3)$ (see Theorem 1.2).
2. Numerical computations suggest that $\mathcal {T}$ is contained in the free algebra $\widetilde{M}[g_1,g_2,g_4,$ $g_6,\ldots ]$. Moreover, it seems that $\mathcal {H}$ is freely generated by $G_2, G_4, G_6$, and $h_k$ for *k* odd.

Given that the partial (respectively, false) Eisenstein-type series $g_k$ (respectively, $h_k$) share many properties with classical Eisenstein series, it is natural to ask whether their Fourier expansions admit a similar shape. We show that this is indeed the case. Recall that the *n*-th Fourier coefficient of the Eisenstein series $G_k$, defined in (1.2), is given by the sum of the $(k-1)$-th powers of the divisors of *n*. In the same spirit, we show that the *n*-th Fourier coefficient of $g_k$ (respectively, $h_k$) can be expressed as an integer linear combination of $(k-1)$-th powers of integers lying in a specified range depending on *n*. To describe these coefficients explicitly, let $\ell (\lambda ):=\sum _{j=1}^n m_j$ denote the *length* of a partition $\lambda$, and define the set^3^

$$\begin{aligned} \Lambda (n,m):=\left\{ \lambda \vdash n \,:\, m_1\ge m_2\ge \cdots \ge m_n\ge 0,\ \ell (\lambda )=m\right\} . \end{aligned}$$

### Theorem 1.5

For $k\in \mathbb {N}$, we have

$$\begin{aligned} g_k(\tau ) = {-\frac{\delta _{k,1}}{2}}+\sum _{n\ge 1} \sum _{m=1}^n a_{n,m} {m^{k-1}} q^n,\ \ \text {and} \ \ T_0(\tau )= 2iq^{\frac{1}{8}}\exp \left( -\sum _{n\ge 1} \sum _{m=1}^n \frac{a_{n,m}}{m} q^n\right) . \end{aligned}$$

Here $a_{n,m}\in \mathbb {Z}$ and $a_{n,m}=0$ if $m<\lceil \tfrac{1}{2} (\sqrt{8n+1}-1) \rceil$. In fact, we have

$$\begin{aligned} a_{n,m}:=\sum _{\lambda \in \Lambda (n,m)} (-1)^{m+m_1}\frac{m}{m_{1}} \left( {\begin{smallmatrix}m_1 \\ \\ m_1-m_2,m_2-m_3,\ldots \end{smallmatrix}}\right) . \end{aligned}$$

We have a similar result for $h_k$ with

$$\begin{aligned}\Omega (n,m):=\left\{ \lambda \vdash n \,:\, m_{3j-2}\ge m_{3j+1}, m_{3j-1}\ge m_{3j+2},\, m_{3j}=0 \text { for all }j\in \mathbb {N},\, 3\ell (\lambda )=m+m_1 \right\} . \end{aligned}$$

### Theorem 1.6

For $k\in \mathbb {N}$, we have

$$\begin{aligned} h_k(\tau )&= -\frac{\delta _{k,1}}{2} + \sum _{n\ge 1} \sum _{m=1}^{2n} b_{n,m} m^{k-1} q^n, \quad \text {and}\quad \eta (\tau )=q^{\frac{1}{24}}\exp \left( -\sum _{n\ge 1} \sum _{m=1}^{2n} \frac{b_{n,m}}{m} q^n\right) \end{aligned}$$

with $b_{n,m}\in \mathbb {Z}$ and $b_{n,m}=0$ if $m< \lfloor \frac{1}{2} (\sqrt{24 n+1}-1)\rfloor$. In fact, we have

$$\begin{aligned} b_{n,m}:=\sum _{\lambda \in \Omega (n,m)} (-1)^{m+m_2}\frac{m}{m_1+m_2}\left( {\begin{smallmatrix}m_1+m_2\\ \\ m_2-m_5,m_5-m_8,\ldots m_1-m_4,m_4-m_7,\ldots \end{smallmatrix}}\right) . \end{aligned}$$

The algebra of functions $g_k$ and $h_k$ can be interpreted in terms of functions coming from unimodal sequences, defined as follows. A *unimodal sequence of size* $n\in \mathbb {N}$ has the form^4^

$$\begin{aligned} 1\le a_1 \le \cdots \le a_{r} \le \underline{c} \ge b_{1} \ge \cdots \ge b_s\ge 1, \end{aligned}$$

for integers $a_1,\ldots ,a_r,c,b_1,\ldots ,b_s$ with $r,s\in \mathbb {N}_0$ and $n=c+\sum _{j=1}^{r}a_j+\sum _{j=1}^{s} b_j$. Let *u*(*n*) count the number of unimodal sequences of size *n*. The *rank* of a unimodal sequence is $s-r$. By convention, the empty sequence is the unique unimodal sequence of size zero; it is of rank zero. Let *u*(*n*, *m*) denote the number of unimodal sequences of size *n* and rank *m*. The generating function for the rank of unimodal sequences [7, (2.2)] is given by ($(a,b)_n:= (a)_n(b)_n$)

$$\begin{aligned} U(\zeta ;q) := \sum _{\begin{array}{c} n\ge 0\\ m\in \mathbb {Z} \end{array}} u(n,m) \zeta ^m q^n = \sum _{n\ge 0} \frac{q^n}{\left( \zeta q, \zeta ^{-1}q\right) _n}. \end{aligned}$$

We look at the exponential Taylor expansion of the unimodal ranks. Namely, we write

1.9 $$\begin{aligned} U(\zeta ;q)&= : \frac{\sin (\pi z)}{\pi z}U(1;q) \exp \left( 2\sum _{k\ge 1} u_k(\tau ) \frac{(2\pi i z)^k}{k!}\right) . \end{aligned}$$

Motivated by the partition Eisenstein traces recently defined by Amdeberhan, Griffin, Ono, and Singh [1], the authors defined in [4], for a sequence of functions $f=\{f_k\}_{k\in \mathbb {N}}$ and $n\in \mathbb {N}_0$, the *n-th partition trace* with respect to *f* and a function $\phi$ on partitions as

$$\begin{aligned} \textrm{Tr}_n(\phi ,f;\tau ){:}{=}\sum _{\lambda \vdash n} \phi (\lambda )f_\lambda (\tau ), \end{aligned}$$

where the sum ranges over all partitions of *n* and, for $\lambda \vdash n$, we set

$$\begin{aligned} f_\lambda (\tau ) := \prod _{j=1}^n f_j^{m_j}(\tau ). \end{aligned}$$

We show that $u_k$ can be recursively expressed as partition traces of the functions $g_k$ and $h_k$. Let

$$\begin{aligned} \mathcal {U}:=\mathcal {H}[g_1,g_2,g_4,g_6,\ldots ]. \end{aligned}$$

### Theorem 1.7

For $k\in \mathbb {N}$, we have $u_k\in \mathcal {U}$. In fact, we have

1.10 $$\begin{aligned} u_k(\tau )&= -\frac{k!}{2} \sum _{\begin{array}{c} \lambda \vdash k\\ \lambda \ne (k^1) \end{array}} \phi (\lambda ) u_\lambda (\tau )+ \textrm{Tr}_k(\phi ,\gamma ;\tau ) + k! g_1(\tau )\textrm{Tr}_{k-1}(\psi ,h;\tau ), \end{aligned}$$

where $u:=\{u_k\}_{k\in \mathbb {N}}, \gamma :=\{G_k-2^{k-1}g_k\}_{k\in \mathbb {N}}, h:=\{h_k\}_{k\in \mathbb {N}}$, and, for $\lambda \vdash k$, we let

$$\begin{aligned} \phi (\lambda ) := \prod _{j=1}^k \frac{2^{m_j}}{j!^{m_j}m_j!},\qquad \psi (\lambda ) := \prod _{j=1}^k \frac{(-1)^{m_j}}{j!^{m_j}m_j!}. \end{aligned}$$

Moreover, the space $\mathcal {U}$ is closed under the action of *D*.

**Remark 1 **Note that $u_k=0$ if *k* is odd, since $U(e^{2\pi i z};q)$ is an even function of *z*. Hence, for *k* odd equation (1.10) gives a relation between $G_\ell , g_\ell$, and $h_\ell$ for $\ell \le k$.

As a direct corollary of Theorems 1.1, 1.2, and 1.7, we recursively give a quasi-completion of $u_k$. More precisely, define the sequence of functions $\widehat{u}:=\{\widehat{u}_k\}_{k\in \mathbb {N}}$ by

$$\begin{aligned} \widehat{u}_k(\tau ,w)&:= -\frac{k!}{2}\sum _{\begin{array}{c} \lambda \vdash k\\ \lambda \ne (k^1) \end{array}} \phi (\lambda ) \widehat{u}_\lambda (\tau ,w)+\frac{k!}{2} \textrm{Tr}_k\left( \phi ,\widehat{\gamma };\tau ,w\right) + k! \widehat{g}_1(\tau ,w)\textrm{Tr}_{k-1}\left( \psi ,\widehat{h};\tau ,w\right) ,\quad \end{aligned}$$

where $\widehat{\gamma }:=\{\widehat{G}_k-2^{k-1}\widehat{g}_k\}_{k\in \mathbb {N}}, \widehat{h}:=\{\widehat{h}_k\}_{k\in \mathbb {N}}$, and $\phi$ and $\psi$ are defined in Theorem 1.7.

### Corollary 1.8

The functions $\widehat{u}_k(\tau ,w)$ transform quasimodular of weight (*k*, 0) for the simultaneous action of $\Gamma _0(3)$ on $\tau$ and *w*. Furthermore, for any $\varepsilon >0$, we have

$$\begin{aligned} \lim _{t\rightarrow \infty } \widehat{u}_k(\tau ,\tau +it+\varepsilon )=u_k(\tau ). \end{aligned}$$

The paper is organized as follows. In Sect. 2, we recall some combinatorial lemmas and some basic properties of the Dedekind eta function, (false) theta functions, and quasimodular forms. In Sect. 3, we prove Theorems 1.1 and 1.2. Next, in Sect. 4, we prove Theorems 1.3 and 1.4. Theorems 1.5 and 1.6 are proven in Sect. 5, and Theorem 1.7 in Sect. 6. In Sect. 7, we present some examples. Finally, in Sect. 8, we raise some questions for future research.

## Preliminaries

### Combinatorial lemmas

We require a lemma regarding the divisibility of multinomial coefficients, which follows directly from Bézout’s Lemma.^5^

#### Lemma 2.1

For $a_1,\ldots ,a_\ell \in \mathbb {N}$ with $\sum _{j=1}^\ell a_j=n$, we have

$$ \frac{n}{\gcd (a_1,\ldots ,a_\ell )} \Big | \left( {\begin{smallmatrix}n\\ \\ a_1,a_2,\ldots ,a_\ell \end{smallmatrix}}\right) . $$

We also need the following elementary lemma.

#### Lemma 2.2

Let $n_1\ge n_2\ge \ldots \ge n_r\ge 0$, $a_1,\ldots ,a_r, b_1,\ldots ,b_r\in \mathbb {R}$, and assume that for all $s\in \{1,\ldots ,r\},$ we have $\sum _{j=1}^s a_j \le \sum _{j=1}^s b_j$. Then

$$ \sum _{j=1}^r a_j n_j \le \sum _{j=1}^r b_j n_j. $$

Next we recall the Faà di Bruno formula which gives ($n\in \mathbb {N}$)

2.1 $$\begin{aligned} \hspace{-0.25cm}{d^{n} \over dz^{n}}f(g(z)) =\sum _{\lambda \vdash n} {\frac{n!}{m_{1}! m_{2}! \cdots m_{n}!}} f^{(m_{1}+\cdots +m_{n})}(g(z)) \prod _{j=1}^{n}\left( {\frac{g^{(j)}(z)}{j!}}\right) ^{m_{j}}. \end{aligned}$$

Finally we use a result on Pólya’s cycle index polynomials [14].

#### Lemma 2.3

We have, as a formal power series in *w*,

$$\begin{aligned} \sum _{{k\ge 0}}\sum _{\lambda \vdash k}\prod _{j=1}^k\frac{x_j^{m_j}}{m_j!}w^k=\exp \left( \sum _{{k\ge 1}}x_k w^k\right) . \end{aligned}$$

### The Dedekind eta function and (false) theta functions

First, we recall the multiplier for the Dedekind eta function,

$$\begin{aligned} \nu _\eta \! \begin{pmatrix}a& b\\ c& d\end{pmatrix}:=\frac{\eta \Bigl (\frac{a\tau +b}{c\tau +d}\Bigr )}{ \sqrt{c\tau +d}\,\eta (\tau )},\qquad \left( \begin{pmatrix} a& b \\ c& d \end{pmatrix}\in \textrm{SL}_2(\mathbb {Z})\right) , \end{aligned}$$

which does not depend on the choice of $\tau$. Then we have the following transformation of $\vartheta$.

#### Lemma 2.4

For  and $m,n\in \mathbb {Z}$, we have

$$\begin{aligned} \vartheta \left( \frac{z}{c\tau +d};\frac{a\tau +b}{c\tau +d}\right)&= \nu _\eta ^3 (\gamma ) \sqrt{c\tau +d} e^{\frac{\pi icz^2}{c\tau +d}} \vartheta (z;\tau ),\\ \vartheta (z+m\tau +n;\tau )&= (-1)^{m+n}q^{-\frac{m^2}{2}}\zeta ^{-m} \vartheta (z;\tau ).\end{aligned}$$

The Jacobi theta function is an example of a Jacobi form.^6^ Another operator for functions transforming like Jacobi forms is the *heat operator* defined, for $m\in \mathbb {Q}$, as

2.2 $$\begin{aligned} H_m:= 4m q\frac{\partial }{\partial q}-\left( \zeta \frac{\partial }{\partial \zeta }\right) ^2. \end{aligned}$$

This operator preserves the elliptic transformation of a Jacobi form of index *m* and in special cases also the modularity, while increasing the weight by two.

Next, consider the false theta function from [3]

$$\begin{aligned} \psi (z;\tau ) := i\sum _{n\in \mathbb {Z}} \operatorname {sgn}\left( n+\frac{1}{2}\right) (-1)^n \zeta ^{n+\frac{1}{2}} q^{\frac{1}{2}\left( n+\frac{1}{2}\right) ^2} \end{aligned}$$

Unlike the Jacobi theta function, $\psi$ is not modular due to the presence of an extra $\operatorname {sgn}$ factor. The first author and Nazaroglu found the following (Jacobi) completion of $\psi$ ($\tau ,w\in \mathbb {H}$ and $z\in \mathbb {C}$)

$$\begin{aligned}\widehat{\psi }(z;\tau ,w) := i\sum _{n\in \mathbb {Z}} \operatorname {erf}\left( -i\sqrt{\pi i(w-\tau )} \left( n+\frac{1}{2}+\frac{z_2}{\tau _2}\right) \right) (-1)^n q^{\frac{1}{2}\left( n+\frac{1}{2}\right) ^2} \zeta ^{n+\frac{1}{2}}, \end{aligned}$$

where $\operatorname {erf}(z):=\frac{2}{\sqrt{\pi }}\int _0^ze^{-t^2}dt$. To recover $\psi$, one needs to take a certain limit. More precisely, we have the following lemma [3, Theorem 2.3 and (1.3)].

#### Lemma 2.5

For  and $m,n\in \mathbb {Z}$, we have

$$\begin{aligned} \widehat{\psi }\left( \frac{z}{c\tau +d};\frac{a\tau +b}{c\tau +d},\frac{aw+b}{cw+d}\right)&= \chi _{\tau ,w} (\gamma ) \nu _\eta ^3 (\gamma ) \sqrt{c\tau +d} e^{\frac{\pi icz^2}{c\tau +d}} \widehat{\psi }(z;\tau ,w),\\ \widehat{\psi }(z+m\tau +n;\tau ,w)&= (-1)^{m+n}q^{-\frac{m^2}{2}}\zeta ^{-m} \widehat{\psi }(z;\tau ,w). \end{aligned}$$

Furthermore, $\psi$ is the holomorphic part of $\widehat{\psi }$ i.e., if $-\tfrac{1}{2}<\tfrac{z_2}{\tau _2}<\tfrac{1}{2}$ and $\varepsilon >0$ arbitrary, we have

2.3 $$\begin{aligned} \lim _{t\rightarrow \infty } \widehat{\psi }(z;\tau ,\tau +it+\varepsilon ) = \psi (z;\tau ). \end{aligned}$$

### Quasimodular forms

Recall Ramanujan’s differential equations

2.4 $$\begin{aligned} D(G_2)&= - 2 G_2^2 + \frac{5}{6}G_4,\quad D(G_4) = -8G_2G_4 + \frac{7}{10}G_6,\quad D\left( G_6\right) = -12G_2G_6 + \frac{400}{7}G_4^2. \end{aligned}$$

We also need the well-known fact that

2.5 $$\begin{aligned} D\left( \textrm{Log}(\eta )\right) =-G_2, \end{aligned}$$

where $\textrm{Log}$ denotes the principal branch of the complex logarithm. The function $G_2$ is an example of a quasimodular form. A function *f* on $\mathbb {H}$ is called a *quasimodular form of weight* *k* *and multiplier* $\nu$ *on* $\Gamma \subset \textrm{SL}_2(\mathbb {Z})$ if there exist holomorphic functions $f_j: \mathbb {H} \rightarrow \mathbb {C}$ for $j \in \{0, \ldots , s\}$, for some $s \in \mathbb {N}_0$ with $f_0 = f$, such that the following hold:

1. We have .
2. As $\tau _2 \rightarrow \infty$, $\smash {(c\tau + d)^{-(k-2j)} f_j( \tfrac{a\tau + b}{c\tau + d})}$ is bounded for $j \in \{0, \ldots , s\}$ and .

A real-analytic function $f(\tau ,w)$ *transforms like a quasimodular form of weight* $(k,\ell )$ *and multiplier* $\nu _{\tau ,w}$ *on* $\Gamma \subset \textrm{SL}_2(\mathbb {Z})$ *under the simultaneous action on* $\tau$ *and* *w* if there exist real-analytic functions $f_{n,m}(\tau ,w)$ for $0\le m\le s_1, 0\le n\le s_2$ for some $s_1,s_2\in \mathbb {N}$ with $f_{0,0}(\tau ,w)= f(\tau ,w)$ such that, for all ,

$$\begin{aligned}f \left( \frac{a\tau +b}{c\tau +d},\frac{aw+b}{cw+d}\right)&=\nu _{\tau ,w} (\gamma )(c\tau +d)^{k}(cw+d)^{\ell }\times \sum _{\begin{array}{c} 0\le n\le s_1\\ 0\le m\le s_2 \end{array}} f_{n,m}(\tau ,w)\left( \frac{c}{c\tau +d}\right) ^n\left( \frac{c}{cw+d}\right) ^m. \end{aligned}$$

## Proofs of Theorems 1.1 and 1.2

In this section, we express *h* in terms of *T* and $\vartheta$, and give the completions of *T* and *h*. First, we note that

3.1 $$\begin{aligned} T(z;\tau )&= \vartheta (z;\tau )+\psi (z;\tau ), \quad h(\zeta ;q) = \frac{i}{2}\left( \zeta ^{\frac{1}{2}}-\zeta ^{-\frac{1}{2}}\right) q^{\frac{1}{8}}\sum _{\pm }\mp \zeta ^{\pm 1} T(3z\pm \tau ;3\tau ). \end{aligned}$$

### Modularity

Using (3.1), we define the completion of *T* and *h* as

3.2 $$\begin{aligned} \widehat{T}(z;\tau ,w):=\widehat{T}(z,\overline{z};\tau ,\overline{\tau },w,\overline{w})&:=\vartheta (z;\tau )+\widehat{\psi }(z;\tau ,w),\end{aligned}$$

3.3 $$\begin{aligned} \widehat{H}(z;\tau ,w):=\widehat{H}(z,\overline{z};\tau ,\overline{\tau },w,\overline{w})&:= \frac{i}{2}\left( \zeta ^{\frac{1}{2}}-\zeta ^{-\frac{1}{2}}\right) q^{\frac{1}{8}}\sum _{\pm }\mp \zeta ^{\pm 1} \widehat{T}(3z\pm \tau ;3\tau ,3w). \end{aligned}$$

We have the following modular transformation of $\widehat{T}$.

#### Proposition 3.1

We have, for , (with $\chi _{\tau ,w}$ is defined in (1.8))

$$\begin{aligned} \widehat{T} \left( \frac{z}{c\tau +d};\frac{a\tau +b}{c\tau +d},\frac{aw+b}{cw+d}\right) = \chi _{\tau ,w} (\gamma ) \nu _\eta ^3 (\gamma )\sqrt{c\tau +d} e^{\frac{\pi icz^2}{c\tau +d}} \widehat{T} \left( \chi _{\tau ,w} (\gamma ) z;\tau ,w\right) . \end{aligned}$$

#### Proof

First we note, by (3.1), using that $z\mapsto \vartheta (z;\tau )$ is odd and $z\mapsto \widehat{\psi }(z;\tau ,w)$ is even,

3.4 $$\begin{aligned} \widehat{T}(-z;\tau ,w)&= -\vartheta (z;\tau )+\widehat{\psi }(z;\tau ,w). \end{aligned}$$

Using (3.1) and Lemmas 2.4 and 2.5, we have

$$\begin{aligned} \widehat{T} \left( \frac{z}{c\tau +d};\frac{a\tau +b}{c\tau +d},\frac{aw+b}{cw+d}\right) = \nu _\eta ^3(\gamma )\sqrt{c\tau +d} e^{\frac{\pi icz^2}{c\tau +d}}\left( \vartheta (z;\tau )+ \chi _{\tau ,w}(\gamma )\widehat{\psi }(z;\tau ,w)\right) .\end{aligned}$$

Recalling that $\chi _{\tau ,w}$ takes value in $\{1,-1\}$ and using (3.2) and (3.4) gives the proposition.$\square$

Next we determine the modular transformation for $\widehat{H}$. We first define

3.5 $$\begin{aligned} \widehat{\mathfrak {H}}(z;\tau ,w)&:=\frac{2i\zeta ^{\frac{1}{2}}q^{\frac{1}{24}}}{1-\zeta }\widehat{H}(z;\tau ,w) \end{aligned}$$

#### Proposition 3.2

We have, for  with $a\equiv \ell \ ( \textrm{mod} \, 3 )$ where $\ell \in \{1,-1\}$,

$$\begin{aligned} \widehat{\mathfrak {H}}\!\left( \frac{z}{c\tau +d};\frac{a\tau +b}{c\tau +d}, \frac{aw+b}{cw+d}\right)&= \nu (\gamma ) \sqrt{c\tau +d} e^{\frac{3\pi icz^2}{c\tau +d}} \widehat{\mathfrak {H}}\!\left( \chi _{\tau ,w}(\gamma ) z;\tau ,w\right) , \end{aligned}$$

where $\chi _{\tau ,w}$ is defined in (1.8) and

$$\begin{aligned} \nu (\gamma ) := (-1)^{b+\frac{a-\ell }{3}} e^{\frac{\pi iab}{3}}\ell \nu _\eta ^3 \begin{pmatrix}a& 3b\\ \frac{c}{3}& d\end{pmatrix} . \end{aligned}$$

#### Proof

To determine the transformation of $\widehat{H}$, we first study $\widehat{T}(3z\pm \tau ;3\tau ,3w)$. Using Proposition 3.1, we have, for ,

3.6 $$\begin{aligned}&\widehat{T}\left( \frac{3z}{c\tau +d}\pm \frac{a\tau +b}{c\tau +d};3\frac{a\tau +b}{c\tau +d},3\frac{aw+b}{cw+d}\right) \nonumber \\&=\chi _{\tau ,w}(\gamma )\, \nu _\eta ^3\! \left( \begin{matrix}a& 3b\\ \frac{c}{3}& d\end{matrix}\right) \sqrt{c\tau +d} e^{\frac{\pi ic \left( 3z\pm (a\tau +b)\right) ^2}{3(c\tau +d)}} \widehat{T} \left( \chi _{\tau ,w}(\gamma ) (3z\pm (a\tau +b));3\tau ,3w\right) . \end{aligned}$$

Now, let

$$\begin{aligned}f_{\pm }(z;\tau ,w):= q^{\frac{1}{6}}\zeta ^{\pm 1} \widehat{T}(3z\pm \tau ;3\tau ,3w). \end{aligned}$$

Writing $a=3r+\ell$ $(r\in \mathbb {Z}, \ell \in \{1,-1\})$ and using Lemmas 2.5, 2.4, and (3.1) gives

$$\begin{aligned}\widehat{T}\left( 3z\pm (a\tau +b);3\tau ,3w\right)&=(-1)^{r+b} q^{-\frac{3r^2}{2}-\ell r} \zeta ^{\mp 3r} \widehat{T}(3z\pm \ell \tau ;3\tau ,3w). \end{aligned}$$

Using this and (3.6), we have

$$\begin{aligned}&f_{\pm } \left( \frac{z}{c\tau +d};\frac{a\tau +b}{c\tau +d},\frac{aw+b}{cw+d}\right) \nonumber \\&\quad =(-1)^{r+b} e^{\frac{\pi iab}{3}} e^{\frac{2\pi i \tau }{6} \pm 2\pi i\ell z + \frac{3\pi ic z^2}{c\tau +d}} \chi _{\tau ,w}(\gamma )\, \nu _\eta ^3\! \left( \begin{matrix}a& 3b\\ \frac{c}{3}& d\end{matrix}\right) \sqrt{c\tau +d}\, \widehat{T}\!\left( \chi _{\tau ,w}(\gamma ) (3z\pm \ell \tau );3\tau ,3w\right) \nonumber \\&\quad = (-1)^{r+b} e^{\frac{\pi iab}{3}} \chi _{\tau ,w}(\gamma )\, \nu _\eta ^3\! \left( \begin{matrix}a& 3b\\ \frac{c}{3}& d\end{matrix}\right) \sqrt{c\tau +d}\, e^{\frac{3\pi i c z^2}{c\tau +d}} f_{\pm \ell \chi _{\tau ,w}(\gamma )}\! \left( \chi _{\tau ,w}(\gamma ) z;\tau ,w\right) . \end{aligned}$$

The claim then follows by using (3.3) and (3.5) to rewrite

$$\begin{aligned} \widehat{\mathfrak {H}}(z;\tau ,w)&= f_{-}(z;\tau ;w)- f_{+}(z;\tau ;w). \end{aligned}$$

$$\square $$

### Modular completions of $g_k$ and $h_k$

Analogous to (1.6) and (1.7), we define

3.7 $$\begin{aligned} \widehat{T}(z;\tau ,w)&=: \widehat{T}(0;\tau ,w)\exp \left( -\sum _{\begin{array}{c} k\ge 1\\ \ell \ge 0 \end{array}}\widehat{g}_{k,\ell }(\tau ,w)\frac{(2\pi iz)^k}{k!}\frac{(2\pi i\overline{z})^\ell }{\ell !}\right) ,\end{aligned}$$

3.8 $$\begin{aligned} \widehat{H}(z;\tau ,w)&=: 2i\sin (\pi z) \left[ \frac{\partial }{\partial e^{2\pi ix}}\widehat{H}(x;\tau ,w)\right] _{x=0} \exp \left( -\sum _{\begin{array}{c} k\ge 1\\ \ell \ge 0 \end{array}}\widehat{h}_{k,\ell }(\tau ,w)\frac{(2\pi iz)^k}{k!}\frac{(2\pi i\overline{z})^\ell }{\ell !}\right) . \end{aligned}$$

Using Proposition 3.1 and (3.7), we obtain the following theorem.

#### Theorem 3.3

For , we have

$$\begin{aligned} \widehat{g}_{k,\ell }\left( \frac{a\tau +b}{c\tau +d},\frac{aw+b}{cw+d}\right)&= \chi _{\tau ,w}^{k+\ell } (\gamma ) (c\tau +d)^k(c\overline{\tau }+d)^\ell \widehat{g}_{k,\ell }(\tau ,w) + \delta _{k,2}\delta _{\ell ,0}\frac{ic}{2\pi }(c\tau +d). \end{aligned}$$

Similarly, using (3.5), Proposition 3.2 and (3.8) we get the following theorem.

#### Theorem 3.4

For , we have

$$\begin{aligned} \widehat{h}_{k,\ell }\left( \frac{a\tau +b}{c\tau +d},\frac{aw+b}{cw+d}\right)&= \chi _{\tau ,w}^{k+\ell } (\gamma ) (c\tau +d)^k(c\overline{\tau }+d)^\ell \widehat{h}_{k,\ell }(\tau ,w) + \delta _{k,2}\delta _{\ell ,0}\frac{3ic}{2\pi }(c\tau +d) . \end{aligned}$$

We next study the limiting behavior of $\widehat{g}_{k,\ell }$ and $\widehat{h}_{k,\ell }$.

#### Proposition 3.5

For $\varepsilon >0$, we have

$$\begin{aligned} \lim _{t\rightarrow \infty } \widehat{g}_{k,\ell }(\tau ,\tau +it+\varepsilon )&= \delta _{\ell ,0} g_k(\tau ),\qquad \lim _{t\rightarrow \infty } \widehat{h}_{k,\ell }(\tau ,\tau +it+\varepsilon ) = \delta _{\ell ,0} h_k(\tau ). \end{aligned}$$

#### Proof

Using (3.1) and (2.3), we have

3.9 $$\begin{aligned} \lim _{t\rightarrow \infty }\widehat{T}(z;\tau ,\tau +it+\varepsilon )&= T(z;\tau ). \end{aligned}$$

Similarly, we get, using (3.1), (3.3), (3.9), and (1.5),

3.10 $$\begin{aligned} \lim _{t\rightarrow \infty }\widehat{H}(z;\tau ,\tau +it+\varepsilon )&= h(\zeta ;q), \lim _{t\rightarrow \infty }\left[ \zeta \frac{\partial }{\partial \zeta }\widehat{H}(z;\tau ,\tau +it+\varepsilon )\right] _{\zeta =1} \!\!=\!-q^{-\frac{1}{24}}\eta (\tau ). \end{aligned}$$

Using (3.7), (3.9), and (1.6), we obtain

3.11 $$\begin{aligned} \lim _{t\rightarrow \infty } \widehat{T}(0;\tau ,\tau +it+\varepsilon )&\exp \left( -\sum _{\begin{array}{c} k\ge 1\\ \ell \ge 0 \end{array}}\widehat{g}_{k,\ell }(\tau ,\tau +it+\varepsilon )\frac{(2\pi iz)^k}{k!}\frac{(2\pi i\overline{z})^\ell }{\ell !}\right) \nonumber \\&\hspace{1cm}= T_0(\tau )\exp \left( -\sum _{k\ge 1}g_k(\tau )\frac{(2\pi iz)^k}{k!}\right) . \end{aligned}$$

Similarly, using (3.8), (3.10), and (1.7), we get

3.12 $$\begin{aligned}&\lim _{t\rightarrow \infty } 2i\sin (\pi z) \left[ \frac{\partial }{\partial e^{2\pi ix}}\widehat{H}(x;\tau ,\tau +it+\varepsilon )\right] _{x=0}\exp \left( -\sum _{\begin{array}{c} k\ge 1\\ \ell \ge 0 \end{array}}\widehat{h}_{k,\ell }(\tau ,\tau +it+\varepsilon )\frac{(2\pi iz)^k}{k!}\frac{(2\pi i\overline{z})^\ell }{\ell !}\right) \nonumber \\&= -2i\sin (\pi z) q^{-\frac{1}{24}}\eta (\tau )\exp \left( -\sum _{k\ge 1}h_k(\tau )\frac{(2\pi iz)^k}{k!}\right) . \end{aligned}$$

Using (3.9) in (3.11) and (3.10) in (3.12), respectively, we obtain

$$\begin{aligned} \sum _{\begin{array}{c} k\ge 1\\ \ell \ge 0 \end{array}}\lim _{t\rightarrow \infty }\widehat{g}_{k,\ell }(\tau ,\tau +it+\varepsilon )\frac{(2\pi iz)^k}{k!}\frac{(2\pi i\overline{z})^\ell }{\ell !}&= \sum _{k\ge 1}g_k(\tau )\frac{(2\pi iz)^k}{k!},\\ \sum _{\begin{array}{c} k\ge 1\\ \ell \ge 0 \end{array}}\lim _{t\rightarrow \infty }\widehat{h}_{k,\ell }(\tau ,\tau +it+\varepsilon )\frac{(2\pi iz)^k}{k!}\frac{(2\pi i\overline{z})^\ell }{\ell !}&= \sum _{k\ge 1}h_k(\tau )\frac{(2\pi iz)^k}{k!}. \end{aligned}$$

Comparing coefficients gives the proposition. $\square$

We are now ready to prove Theorems 1.1 and 1.2.

#### Proofs of Theorems 1.1 and 1.2

We define $\widehat{g}_k(\tau ,w):=\widehat{g}_{k,0}(\tau ,w)$ and $\widehat{h}_k(\tau ,w):=\widehat{h}_{k,0}(\tau ,w)$. Then parts (1) of both theorems follow from Theorems 3.3 and 3.4. Parts (2) of the theorems can be concluded from Proposition 3.5. $\square$

## Proofs of Theorems 1.3 and 1.4

In this section, we show that *T* and *h* are eigenforms of the heat operators.

### Proof of Theorem 1.3

A direct calculation shows that *T* is annihilated by the heat operator $H_{\frac{1}{2}}$ defined in (2.2).

#### Lemma 4.1

We have

$$\begin{aligned}H_{\frac{1}{2}}\left( T(z;\tau )\right)&= 0. \end{aligned}$$

#### Proof of Theorem 1.3

It suffices to prove the formulas for $D(g_k)$ and $D(\textrm{Log}(T_0))$. From (1.6),

4.1 $$\begin{aligned}&q\frac{\partial }{\partial q}T(z;\tau ) = T(z;\tau )\left( q\frac{\partial }{\partial q}\textrm{Log}\left( T_0(\tau )\right) -\sum _{k\ge 1} q\frac{\partial }{\partial q}g_k(\tau )\frac{(2\pi iz)^k}{k!}\right) . \end{aligned}$$

Furthermore, we have

4.2 $$\begin{aligned}&\left( \zeta \frac{\partial }{\partial \zeta }\right) ^2 T(z;\tau ) = T(z;\tau )\left( -\sum _{k\ge 1} k(k-1)g_k(\tau )\frac{(2\pi iz)^{k-2}}{k!} + \left( \sum _{k\ge 1}kg_k(\tau )\frac{(2\pi iz)^{k-1}}{k!}\right) ^{ 2} \right) . \end{aligned}$$

Using Lemma 4.1, (4.1), and (4.2) gives

$$\begin{aligned} H_{\frac{1}{2}}(T(z;\tau ))&=T(z;\tau ) \left( 2\left( q\frac{\partial }{\partial q}\textrm{Log}\left( T_0(\tau )\right) -\sum _{k\ge 1} q\frac{\partial }{\partial q}g_k(\tau )\frac{(2\pi iz)^k}{k!}\right) \right. \\&\qquad \left. +\sum _{k\ge 1} k(k-1)g_k(\tau )\frac{(2\pi iz)^{k-2}}{k!} - \left( \sum _{k\ge 1}kg_k(\tau )\frac{(2\pi iz)^{k-1}}{k!}\right) ^{ 2} \right) =0. \end{aligned}$$

Since $T(z;\tau )\ne 0$, this is equivalent to

$$\begin{aligned} 2\sum _{k\ge 1} q\frac{\partial }{\partial q}g_k(\tau )\frac{(2\pi iz)^k}{k!}&= \sum _{k\ge 0}g_{k+2}(\tau )\frac{(2\pi iz)^{k}}{k!} - \left( \sum _{k\ge 0}g_{k+1}(\tau )\frac{(2\pi iz)^{k}}{k!}\right) ^{ 2} \\&\quad + 2q\frac{\partial }{\partial q}\textrm{Log}\left( T_0(\tau )\right) . \end{aligned}$$

Comparing the coefficients of $(2\pi iz)^k$ gives the theorem. $\square$

### Proof of Theorem 1.4

We show that a multiple of *h* is annihilated by the heat operator. More precisely, define

4.3 $$\begin{aligned} \mathfrak {H}(z;\tau ):=\frac{2iq^{\frac{1}{24}}\zeta ^{\frac{1}{2}}h(\zeta ;q)}{1-\zeta }. \end{aligned}$$

#### Lemma 4.2

We have

$$ H_{\frac{3}{2}} (\mathfrak {H}(z;\tau ) ) = 0. $$

#### Proof

From (1.5), we obtain

4.4 $$\begin{aligned} \mathfrak {H}(z;\tau ) = 2i\sum _{n\ge 0} (-1)^n \zeta ^{3n+\frac{1}{2}} q^{\frac{1}{6}\left( 3n+\frac{1}{2}\right) ^2} - 2i\sum _{n\ge 0} (-1)^n \zeta ^{3n+\frac{5}{2}} q^{\frac{1}{6}\left( 3n+\frac{5}{2}\right) ^2}. \end{aligned}$$

We have that

$$\begin{aligned} H_{\frac{3}{2}}\!\left( \zeta ^{3n+\frac{1}{2}} q^{\frac{1}{6}\left( 3n+\frac{1}{2}\right) ^2}\right) = H_{\frac{3}{2}}\!\left( \zeta ^{3n+\frac{5}{2}} q^{\frac{1}{6}\left( 3n+\frac{5}{2}\right) ^2} \right) =0. \end{aligned}$$

Plugging these into (4.4) gives the claim. $\square$

#### Proof of Theorem 1.4

By (4.3) and (1.7), we have

4.5 $$\begin{aligned} \mathfrak {H}(z;\tau ) = 2i\eta (\tau )\exp \left( -\sum _{k\ge 1}h_k(\tau )\frac{(2\pi iz)^k}{k!}\right) . \end{aligned}$$

Using this, we compute

4.6 $$\begin{aligned}&q\frac{\partial }{\partial q} \mathfrak {H}(z;\tau )= \mathfrak {H}(z;\tau ) \left( q\frac{\partial }{\partial q} \textrm{Log}\left( \eta (\tau )\right) -\sum _{k\ge 1}q\frac{\partial }{ \partial q}h_k(\tau )\frac{(2\pi iz)^k}{k!}\right) . \end{aligned}$$

Next, we have

4.7 $$\begin{aligned} \hspace{-3pt}\left( \zeta \frac{\partial }{\partial \zeta }\right) ^{ 2} \mathfrak {H}(z;\tau ) = \mathfrak {H}(z;\tau ) \left( \left( \sum _{k\ge 0}h_{k+1}(\tau )\frac{(2\pi iz)^{k}}{k!}\right) ^{ 2} -\sum _{k\ge 0}h_{k+2}(\tau )\frac{(2\pi iz)^{k}}{k!} \right) . \end{aligned}$$

Plugging (4.6), (4.7), and (4.5) into Lemma 4.2 then gives

$$\begin{aligned} H_{\frac{3}{2}}\left( \mathfrak {H}(z;\tau ) \right)&= \left( 6q\frac{\partial }{\partial q} \textrm{Log}\left( \eta (\tau )\right) -6\sum _{k\ge 1}q\frac{\partial }{ \partial q}h_k(\tau )\frac{(2\pi iz)^k}{k!} +\sum _{k\ge 0}h_{k+2}(\tau )\frac{(2\pi iz)^{k}}{k!} \right. \\&\hspace{2.5cm}\left. - \left( \sum _{k\ge 0}h_{k+1}(\tau )\frac{(2\pi iz)^{k}}{k!}\right) ^{ 2}\right) \mathfrak {H}(z;\tau )=0. \end{aligned}$$

Since $\mathfrak H(z;\tau )\ne 0$, by comparing the coefficient of $(2\pi iz)^k$ for $k\in \mathbb {N}$, we get

4.8 $$\begin{aligned} 6q\frac{\partial }{\partial q}h_k(\tau )&= h_{k+2}(\tau ) -\sum _{d=0}^k \left( {\begin{smallmatrix}k\\ \\ d\end{smallmatrix}}\right) h_{d+1}(\tau )h_{k-d+1}(\tau ). \end{aligned}$$

Furthermore, comparing the constant term and using (2.5) gives

4.9 $$\begin{aligned} -G_2(\tau ) = q\frac{\partial }{\partial q} \textrm{Log}\left( \eta (\tau )\right)&= -\frac{h_2(\tau )}{6} + \frac{h_1^2(\tau )}{6}. \end{aligned}$$

It remains to show that $\mathcal {H} =\widetilde{\mathcal {M}}[h_1,h_3,h_5,\ldots ]$. Using (4.9), (4.8), and (2.4), we have $G_2,G_4,G_6\in \mathcal {H}=\mathbb {C}[h_1,h_2,\ldots ]$. To finish the proof, we need to show that $h_k$ for *k* even can be written as a polynomial in $G_2,G_4,G_6$, and $h_j$ with *j* odd. This follows inductively by differentiating (4.9) and using (4.8) and the fact that the space of quasimodular forms is closed under differentiating. $\square$

## Proofs of Theorems 1.5 and 1.6

In this section, we give explicit formulas for the Fourier coefficients of $g_k$ and $h_k$.

### Constant terms

We first compute the constant terms of $g_k$ and $h_k$.

#### Lemma 5.1

We have

$$\begin{aligned} \lim _{\tau \rightarrow i\infty } g_k(\tau ) = \lim _{\tau \rightarrow i\infty }h_k(\tau ) = -\frac{\delta _{k,2}}{2}. \end{aligned}$$

#### Proof

From (1.6) and (1.7), we have

$$\begin{aligned} \frac{T(z;\tau )}{T_0(\tau )}&= \exp \left( -\sum _{k\ge 1}g_k(\tau )\frac{(2\pi iz)^k}{k!}\right) , \\ \frac{iq^{\frac{1}{24}}h(\zeta ;q)}{2\sin (\pi z)\eta (\tau )}&= \exp \left( -\sum _{k\ge 1}h_k(\tau )\frac{(2\pi iz)^k}{k!}\right) . \end{aligned}$$

Using (1.4) and (1.5), note that

$$\begin{aligned} T(z;\tau ) = 2i\zeta ^{\frac{1}{2}}q^{\frac{1}{8}}(1+O_\zeta (q)),\qquad h(\zeta ;q)=1-\zeta + O_\zeta (q),\qquad q^{-\frac{1}{24}}\eta (\tau )=1+O(q). \end{aligned}$$

Hence we have

$$\begin{aligned} \exp \left( -\sum _{k\ge 1} \lim _{\tau \rightarrow i\infty }g_k(\tau )\frac{(2\pi iz)^k}{k!}\right)&= \lim _{\tau \rightarrow i\infty } \frac{T(z;\tau )}{T_0(\tau )}=\zeta ^{\frac{1}{2}}=\exp \left( \frac{1}{2}\frac{2\pi i z}{1!}\right) ,\\ \exp \left( -\sum _{k\ge 1} \lim _{\tau \rightarrow i\infty }h_k(\tau )\frac{(2\pi iz)^k}{k!}\right)&= \lim _{\tau \rightarrow i\infty }\frac{ih(\zeta ;q)}{2\sin (\pi z)q^{-\frac{1}{24}}\eta (\tau )} = \frac{\zeta -1}{2 i \sin (\pi z)}= \zeta ^{\frac{1}{2}}\\&=\exp \left( \frac{1}{2} \frac{2\pi i z}{1!}\right) . \end{aligned}$$

Taking the logarithm of these and comparing coefficients gives the lemma. $\square$

### Proof of Theorem 1.5

#### Proof of Theorem 1.5

From (1.6), we have,

5.1 $$\begin{aligned} \sum _{k\ge 0}g_k(\tau )\frac{(2\pi iz)^k}{k!}=-\textrm{Log}\left( \texttt{T}(\zeta ;q)\right) \end{aligned}$$

with $$g_0(\tau ):=-\textrm{Log}\left( \texttt{T}(1;q)\right) $$ and

5.2 $$\begin{aligned} \texttt{T}(\zeta ;q):= \frac{T(z;\tau )}{2iq^{\frac{1}{8}}}= \sum _{n\ge 0} (-1)^n \zeta ^{n+\frac{1}{2}} q^{\frac{n(n+1)}{2}}. \end{aligned}$$

Applying (2.1) to $f(q)=\textrm{Log}(q)$ and $g(q)=\texttt{T}(\zeta ;q)$, we obtain, for $n\in \mathbb {N}$, that

$$\begin{aligned} \hspace{-0.15cm}{\partial ^{n} \over \partial q^{n}}\textrm{Log}(\texttt{T}(\zeta ;q))&=\sum _{\lambda \vdash n} \frac{n!\big (\sum _{r=1}^n m_r -1\big )!(-1)^{\sum _{r=1}^n m_r +1}}{m_{1}!m_{2}!\cdots m_{n}!\texttt{T}(\zeta ;q)^{\sum _{r=1}^n m_r}} \prod _{j=1}^{n}\left( \frac{\frac{\partial ^j}{\partial q^j}\texttt{T}(\zeta ;q)}{j!}\right) ^{m_{j}} .\end{aligned}$$

Note that from (5.2) $\texttt{T}(\zeta ;0)=\zeta ^\frac{1}{2}$. Dividing by *n*! and inserting the definition of $\texttt{T}$, we obtain

$$\begin{aligned}&\operatorname {coeff}_{[q^n]}\textrm{Log}(\texttt{T}(\zeta ;q)) \\&=\lim \limits _{q\rightarrow 0^+}\sum _{\lambda \vdash n} {\frac{(-1)^{\sum _{r=1}^n m_r +1}}{\sum _{j=1}^n m_j}}\zeta ^{-\frac{1}{2}\sum _{r=1}^nm_r}\left( {\begin{smallmatrix}\sum _{r=1}^n m_r \\ \\ m_1,m_2,\ldots ,m_n\end{smallmatrix}}\right) \prod _{j=1}^{n}\left( \frac{\frac{\partial ^j}{\partial q^j}\sum _{n\ge 0} (-1)^n \zeta ^{n+\frac{1}{2}} q^{\frac{n(n+1)}{2}}}{j!}\right) ^{m_{j}}. \end{aligned}$$

Let $T_\ell :=\frac{\ell (\ell +1)}{2}$ be the $\ell$*-th triangular number* and denote by $\mathscr {T}(n)$ the set of all partitions of *n* into triangular numbers. For a partition $\lambda \in \mathscr {T}(n)$, we write $\lambda = ( T_1^{m_{T_1}}, T_2^{m_{T_2}},\ldots )$. Noting that $\sum _{r=1}^n m_r = \sum _{\ell \ge 1} m_{T_\ell }$, we have

5.3 $$\begin{aligned} \operatorname {coeff}_{[q^n]}\textrm{Log}(\texttt{T}(\zeta ;q))&=\sum _{\lambda \in \mathscr {T}(n)} \frac{(-1)^{\sum _{\ell \ge 1} m_{T_\ell } +1}\zeta ^{-\frac{1}{2}\sum _{\ell \ge 1} m_{T_\ell }}}{\sum _{\ell \ge 1} m_{T_\ell }} \left( {\begin{smallmatrix}\sum _{\ell \ge 1} m_{T_\ell } \\ \\ m_{T_1},m_{T_2},\ldots \end{smallmatrix}}\right) \prod _{\ell \ge 1}\left( (-1)^\ell \zeta ^{\ell +\frac{1}{2}}\right) ^{m_{T_\ell }} \nonumber \\&=\sum _{\lambda \in \mathscr {T}(n)} \frac{(-1)^{\sum _{\ell \ge 1} m_{T_\ell } +1}}{\sum _{\ell \ge 1} m_{T_\ell }}\left( {\begin{smallmatrix}\sum _{\ell \ge 1} m_{T_\ell } \\ \\ m_{T_1},m_{T_2},\ldots \end{smallmatrix}}\right) \left( -\zeta \right) ^{\sum _{\ell \ge 1}\ell m_{T_\ell }} . \end{aligned}$$

Let $\mathscr {G}(n)$ be the set of partitions with non-increasing multiplicities i.e., $\lambda =(1^{m_1},2^{m_2},\ldots ,n^{m_n})\vdash n$ with $m_1\ge m_2\ge \ldots \ge m_n \ge 0$. We define a bijection $\mathscr {T}(n)\rightarrow \mathscr {G}(n)$

$$\begin{aligned} \left( T_1^{m_{T_1}}, T_2^{m_{T_2}},T_3^{m_{T_3}},\ldots \right) \mapsto \left( 1^{\ell (\lambda )},2^{\ell (\lambda )-m_{T_1}},3^{\ell (\lambda )-m_{T_1}-m_{T_2}},\ldots \right) \end{aligned}$$

by writing each part $T_\ell$ of $\lambda$ as $T_\ell =\sum _{j=1}^{\ell } j$. The inverse map is given by

$$\begin{aligned}&\left( 1^{m_1},2^{m_2},\ldots , n^{m_n} \right) \mapsto \left( T_1^{m_1-m_2}, T_2^{m_2-m_3},\ldots \right) . \end{aligned}$$

Hence, (5.3) simplifies as

5.4 $$\begin{aligned} \operatorname {coeff}_{[q^n]}\textrm{Log}(\texttt{T}(\zeta ;q)) =\sum _{\lambda \in \mathscr {G}(n)} {\frac{(-1)^{m_1 +1}}{m_{1}}}\left( {\begin{smallmatrix}m_1 \\ \\ m_1-m_2,m_2-m_3,\ldots \end{smallmatrix}}\right) (-\zeta )^{\sum _{j\ge 1}m_j} . \end{aligned}$$

Using (5.1), Lemma 5.1, and (5.4), we have

$$\begin{aligned} g_k(\tau )&= -\frac{\delta _{k,1}}{2}-{k!}\operatorname {coeff}_{\left[ (2\pi iz)^k\right] }\sum _{n\ge 1}\sum _{\lambda \in \mathscr {G}(n)} {\frac{(-1)^{m_1 +1}}{m_{1}}}\left( {\begin{smallmatrix}m_1 \\ \\ m_1-m_2,m_2-m_3,\ldots \end{smallmatrix}}\right) \left( -\zeta \right) ^{\sum _{j\ge 1} m_j} q^n\\&=-\frac{\delta _{k,1}}{2}+\sum _{n\ge 1} \sum _{\lambda \in \mathscr {G}(n)} {\frac{(-1)^{\sum _{j\ge 2} m_j}}{m_{1}}}\left( {\begin{smallmatrix}m_1 \\ \\ m_1-m_2,m_2-m_3,\ldots \end{smallmatrix}}\right) \sum _{j\ge 1}m_j \left( \sum _{j\ge 1} m_j\right) ^{k-1} q^{n}. \end{aligned}$$

Note that, using Lemma 2.1, we have

$$ {\frac{(-1)^{\sum _{j\ge 2}m_j}}{m_{1}}} \sum _{j\ge 1}m_j \left( {\begin{smallmatrix}m_1 \\ \\ m_1-m_2,m_2-m_3,\ldots \end{smallmatrix}}\right) \in \mathbb {Z}. $$

Hence, we have, with $a_{n,m}\in \mathbb {Z}$ as in the theorem,

$$\begin{aligned}g_k(\tau ) = {-\frac{\delta _{k,1}}{2}}+\sum _{n\ge 1} \sum _{m=1}^n a_{n,m} {m^{k-1}} q^n. \end{aligned}$$

We claim that the conditions in $\Lambda (n,m)$ give

$$\begin{aligned}m=\sum _{j=1}^n m_j \ge \frac{1}{2}\left( \sqrt{8n+1}-1\right) . \end{aligned}$$

Indeed, let $\ell \in \mathbb {N}$ be the unique integer such that $T_\ell \le n<T_{\ell +1}$. Recall the constraints $m_1 \ge m_2 \ge \cdots \ge m_n \ge 0$, and $\sum _{j=1}^n j m_j = n.$ Hence, if $m_{\ell +1}\ge 1$, we find $m_1, \ldots ,m_{\ell +1}\ge 1$ and therefore $n=\sum _{j=1}^n j m_j \ge \sum _{j=1}^{\ell +1} j = T_{\ell +1}>n$, a contradiction. We conclude that $m_{\ell +1}=0$. Furthermore, we also have that $0\le m_\ell \le 1$, since otherwise $m_1\ge m_2\ge \cdots \ge m_\ell \ge 2$ which gives $n=\sum _{j=1}^\ell jm_j\ge 2\sum _{j=1}^\ell j=2T_\ell$, again a contradiction. Using Lemma 2.2 with $n_j=m_j$, $a_j=j,$ and $b_j=\frac{\ell }{2}$ for $j\in \{1,\ldots ,\ell -1\}$ as well as $m_\ell \le 1$, we find that

5.5 $$\begin{aligned} n&\le \frac{\ell }{2}m +\frac{\ell }{2}, \qquad \text {or equivalently} \qquad m+1\ge \frac{2n}{\ell }. \end{aligned}$$

As $T_\ell \le n$ (and $\ell >0$), we find $\ell \le \frac{1}{2}(\sqrt{8n+1}-1)$. Plugging this into (5.5) gives $m\ge \frac{1}{2} (\sqrt{8 n+1}-1)$. Thus $a_{n,m}=0$ unless $\frac{1}{2}(\sqrt{8n+1}-1) \le m \le n$. This proves the theorem. $\square$

### Proof of Theorem 1.6

#### Proof of Theorem 1.6

Using (1.7), we have

5.6 $$\begin{aligned} \sum _{k\ge 0}h_k(\tau )\frac{(2\pi iz)^k}{k!}&= -\textrm{Log}\left( \texttt{H}(\zeta ;q)\right) \end{aligned}$$

with $$h_0(\tau ):=-\textrm{Log}(q^{-\frac{1}{24}}\eta (\tau ))$$, and

5.7 $$\begin{aligned} \texttt{H}(\zeta ;q):= \frac{ih(\zeta ;q)}{2\sin (\pi z)}. \end{aligned}$$

Applying (2.1) with $f(q)=\textrm{Log}(q)$ and $g(q)=\texttt{H}(\zeta ;q)$, we obtain, for $n\in \mathbb {N}_0$,

5.8 $$\begin{aligned} {\partial ^{n} \over \partial q^{n}}\textrm{Log}\left( \texttt{H}(\zeta ;q)\right) =\sum _{\lambda \vdash n} \frac{(-1)^{\sum _{r=1}^n m_r +1}n! \big (\sum _{r=1}^n m_r -1\big )!}{m_{1}! m_{2}! \cdots m_{n}! \texttt{H}(\zeta ;q)^{\sum _{r=1}^n m_r}} \prod _{j=1}^{n}\left( \frac{\frac{\partial ^j}{\partial q^j}\texttt{H}(\zeta ;q)}{j!}\right) ^{m_{j}} . \end{aligned}$$

Using (1.5) and (5.7), we get

$$\begin{aligned} \texttt{H}(\zeta ;q)&= \zeta ^{\frac{1}{2}} \sum _{n\ge 0} (-1)^n \zeta ^{3n} q^{\frac{n(3n+1)}{2}} + \zeta ^{-\frac{1}{2}}\sum _{n\ge 1} (-1)^n \zeta ^{3n} q^{\frac{n(3n-1)}{2}}. \end{aligned}$$

From this, we obtain that

$$\begin{aligned} \left[ \frac{\partial ^j}{\partial q^j}\texttt{H}(\zeta ;q)\right] _{q=0}&= {\left\{ \begin{array}{ll} \zeta ^{\frac{1}{2}} &  \text {if }j=0,\\ (-1)^{\ell }\zeta ^{3\ell -\frac{1}{2}} P_{\ell }! & \text {if } j=P_{\ell } \text { with }\ell \in \mathbb {N},\\ (-1)^{\ell }\zeta ^{3\ell +\frac{1}{2}} P_{-\ell }! &  \text {if } j=P_{-\ell } \text { with } \ell \in \mathbb {N},\\ 0 & \text {otherwise}, \end{array}\right. } \end{aligned}$$

where for $\ell \in \mathbb {Z}\setminus \{0\}$, we let $P_{\ell }:=\tfrac{\ell (3\ell - 1)}{2}$ be the $\ell$*–th generalized pentagonal number*. Moreover, denote by $\mathscr {P}(n)$ the set of partitions of *n* into generalized pentagonal numbers. We write a partition $\lambda \in \mathscr {P}(n)$ as $\lambda =(P_{1}^{m_{P_{1}}},P_{-1}^{m_{P_{-1}}},P_{2}^{m_{P_{2}}},P_{-2}^{m_{P_{-2}}},\ldots )$. Note that we have $\sum _{r=1}^n m_r=\sum _{\ell \ge 1} (m_{P_\ell }+m_{P_{-\ell }})$. With this notation, (5.8) becomes

5.9 $$\begin{aligned} \operatorname {coeff}_{[q^n]}\textrm{Log}\left( \texttt{H}(\zeta ;q)\right)&=\sum _{\lambda \in \mathscr {P}(n)} \frac{(-1)^{\sum _{\ell \ge 1} \left( m_{P_\ell }+m_{P_{-\ell }}\right) +1}}{\sum _{\ell \ge 1} \left( m_{P_\ell }+m_{P_{-\ell }}\right) }\left( {\begin{smallmatrix}\sum _{\ell \ge 1} \left( m_{P_\ell }+m_{P_{-\ell }}\right) \\ \\ m_{P_1},m_{P_{-1}},m_{P_2},m_{P_{-2}},\ldots \end{smallmatrix}}\right) \nonumber \\&\qquad \times \zeta ^{-\frac{1}{2}\sum _{\ell \ge 1} \left( m_{P_\ell }+m_{P_{-\ell }}\right) } \prod _{\ell \ge 1}\left( (-1)^{\ell }\zeta ^{3\ell -\frac{1}{2}}\right) ^{m_{P_{\ell }}} \prod _{\ell \ge 1} \left( (-1)^{\ell } \zeta ^{3\ell +\frac{1}{2}}\right) ^{m_{P_{-\ell }}} \nonumber \\&\hspace{-3cm}=\sum _{\lambda \in \mathscr {P}(n)} \frac{(-1)^{\sum _{\ell \ge 1}(\ell +1) \left( m_{P_{\ell }}+m_{P_{-\ell }}\right) +1}}{\sum _{\ell \ge 1} (m_{P_\ell }+m_{P_{-\ell }})}\left( {\begin{smallmatrix}\sum _{\ell \ge 1} \left( m_{P_\ell }+m_{P_{-\ell }}\right) \\ \\ m_{P_1},m_{P_{-1}},m_{P_2},m_{P_{-2}},\ldots \end{smallmatrix}}\right) \zeta ^{3\sum _{\ell \ge 1} \ell \left( m_{P_{\ell }}+m_{P_{-\ell }}\right) - \sum _{\ell \ge 1} m_{P_{\ell }}} . \end{aligned}$$

Next, denote by $\mathscr {H}(n)$ the set of partitions of *n* having no parts that are multiple of three and such that $m_{3j-2}\ge m_{3j+1}$ and $m_{3j-1}\ge m_{3j+2}$ for $j\ge 1$. Define a bijection $\mathscr {P}(n)\rightarrow \mathscr {H}(n)$ by

$$ \left( P_{1}^{m_{P_{1}}},P_{-1}^{m_{P_{-1}}},P_{2}^{m_{P_{2}}},P_{-2}^{m_{P_{-2}}},\ldots \right) \mapsto \left( 1^{\sum _{\ell \ge 1} m_{P_\ell }}, 2^{\sum _{\ell \ge 1} m_{P_{-\ell }}}, 4^{\sum _{\ell \ge 2} m_{P_\ell }}, 5^{\sum _{\ell \ge 2} m_{P_{-\ell }}},\ldots \right) , $$

where we write for each part $P_{\ell }=\sum _{m=1}^\ell (3m-2)$ and $P_{-\ell }=\sum _{m=1}^\ell (3m-1)$ for $1\le \ell \le n$. The inverse map is given by

$$\begin{aligned} \left( 1^{m_1},2^{m_2},4^{m_4},5^{m_5},\ldots \right) \mapsto \left( P_1^{m_1-m_4}, P_{-1}^{m_2-m_5}, P_{2}^{m_4-m_7}, P_{-2}^{m_5-m_8},\ldots \right) . \end{aligned}$$

Therefore (5.9) becomes

5.10 $$\begin{aligned} \operatorname {coeff}_{[q^n]}\textrm{Log}\left( \texttt{H}(\zeta ;q)\right)&= \sum _{\lambda \in \mathscr {H}(n)} \frac{(-1)^{\sum _{\ell \ge 1} (m_{3\ell -1}+m_{3\ell -2}) + m_1+m_2+1}}{m_1+m_2} \left( {\begin{smallmatrix}m_1+m_2\\ \\ m_1-m_4,m_2-m_5,\ldots \end{smallmatrix}}\right) \nonumber \\&\qquad \times \zeta ^{3\sum _{\ell \ge 1} (m_{3\ell -1}+m_{3\ell -2})-m_1} . \end{aligned}$$

By Lemma 5.1, (5.6), and (5.10), we have, for $k\in \mathbb {N}$,

$$\begin{aligned} h_k(\tau )&= -\frac{\delta _{k,1}}{2}+ \sum _{n\ge 1} \sum _{\lambda \in \mathscr {H}(n)} \!(-1)^{\sum _{\ell \ge 2} (m_{3\ell -1}+m_{3\ell -2})}\quad \frac{3\sum _{\ell \ge 1} (m_{3\ell -1}+m_{3\ell -2})-m_1}{m_1\!+\!m_2}\left( {\begin{smallmatrix}m_1+m_2\\ \\ m_1-m_4,m_2-m_5,\ldots \end{smallmatrix}}\right) \\&\hspace{1cm}\times \left( 3\sum _{\ell \ge 1} (m_{3\ell -1}+m_{3\ell -2})-m_1\right) ^{\!k-1}\! q^n. \end{aligned}$$

Now let $d:=\gcd (m_1-m_4,m_2-m_5,\ldots )$. By [4, Lemma 2.8], we have $$\frac{m_1+m_2}{d}|\left( {\begin{smallmatrix}m_1+m_2\\ \\ m_1-m_4,m_2-m_5,\ldots \end{smallmatrix}}\right) $$. Moreover, note that *d* divides

$$\begin{aligned}&3\left( m_1-m_4+2(m_4-m_7)+3(m_7-m_{10})+\ldots \right) +3\left( m_2-m_5+2(m_5-m_8)+3(m_8-m_{11})+\ldots \right) \\&\quad - \left( m_1-m_4+m_4-m_7+\ldots \right) = 3\sum _{\ell \ge 1} (m_{3\ell -1}+m_{3\ell -2})-m_1. \end{aligned}$$

Thus, $m_1+m_2=\frac{m_1+m_2}{d}d$ divides $$(3\sum _{\ell \ge 1} (m_{3\ell -1}+m_{3\ell -2})-m_1)\left( {\begin{smallmatrix}m_1+m_2\\ \\ m_1-m_4,m_2-m_5,\ldots \end{smallmatrix}}\right) $$. Hence we get

$$\begin{aligned}h_k(\tau )&= -\frac{\delta _{k,1}}{2} + \sum _{n,m\ge 1} b_{n,m} m^{k-1} q^n, \end{aligned}$$

where $b_{n,m}\in \mathbb {Z}$ is defined as in the theorem.

To finish the proof, we have to show that

5.11 $$\begin{aligned} \left\lfloor \frac{1}{2} \left( \sqrt{24 n+1}-1\right) \right\rfloor \le m\le 2n. \end{aligned}$$

The definition of $\Omega (n,m)$ directly gives that $m\le 2n$. Next we show the lower bound for *m*. It is not hard to see that for fixed *n*, there exists a unique $\ell \in \mathbb {N}_0$ such that either $P_{-\ell }\le n<P_{\ell +1}$ (which we refer to as (1)) or $P_{\ell +1}\le n<P_{-\ell -1}$ (which we refer to as (2)). Recall that (by the condition in $\Omega (n,m)$) $m_1\ge m_4\ge m_7 \ge \ldots \ge 0$, $m_2\ge m_5\ge m_8 \ge \ldots \ge 0$ and $\sum _{j=1}^n jm_j=n$. If $m_{3\ell +4}\ge 1$, then

$$ n=\sum _{j=1}^n jm_j\ge \sum _{j=1}^{\ell +2} (3j-2)m_{3j-2}\ge \sum _{j=1}^{\ell +2} (3j-2) =P_{\ell +2}>P_{-\ell -1}>n, $$

a contradiction. Similarly, if $m_{3\ell +2}\ge 1$, then

$$ n=\sum _{j=1}^n jm_j\ge \sum _{j=1}^{\ell +1} (3j-1)m_{3j-1}\ge \sum _{j=1}^{\ell +1} (3j-1)=P_{-\ell -1}>n, $$

again a contradiction. Finally, if $m_{3\ell +1}\ge 1$ then

$$ n=\sum _{j=1}^n jm_j\ge \sum _{j=1}^{\ell +1} (3j-2)m_{3j-2}\ge \sum _{j=1}^{\ell } (3j-2)=P_{\ell +1}, $$

which is a contradiction if we are in case (1). This gives that $m_j=0$ for all $j\ge 3\ell +2$ in case (2) and for all $j\ge 3\ell +1$ in case (1). Applying Lemma 2.2 with $n_j=m_{3j-1}, a_j=3j-1$, and $b_j=\frac{3\ell +1}{2}$, we obtain

5.12 $$\begin{aligned} \sum _{j=1}^{\ell } (3j-1)m_{3j-1} \le \frac{3\ell +1}{2}\sum _{j=1}^\ell m_{3j-1}. \end{aligned}$$

Similarly applying Lemma 2.2 with $r=\ell +1$, $n_j=m_{3j-2}$, $a_j=3j-2$, and $b_j=\frac{\ell }{2}(3-\delta _{j,1})$ (resp. $b_j = \frac{\ell +1}{2}(3-\delta _{j,1})$) if we are in case (1) (resp. (2)), we get

5.13 $$\begin{aligned} \sum _{j=1}^{\ell +1} (3j-2)m_{3j-2} \le {\left\{ \begin{array}{ll} \frac{3\ell }{2}\left( \frac{2}{3}m_1+\sum _{j=2}^{\ell +1} m_{3j-2}\right) &  \hspace{-0.2cm}\text {if we are in case (1)}, \\ \frac{3\ell +3}{2}\left( \frac{2}{3}m_1+\sum _{j=2}^{\ell +1} m_{3j-2}\right) &  \hspace{-0.2cm}\text {if we are in case (2)}. \end{array}\right. } \end{aligned}$$

By combining (5.12) and (5.13), we deduce

5.14 $$\begin{aligned} n&=\sum _{j=1}^{3\ell +1} j m_j = \sum _{j=1}^{\ell } (3j-1)m_{3j-1} + \sum _{j=1}^{\ell +1} (3j-2)m_{3j-2} \le {\left\{ \begin{array}{ll} \frac{(3\ell +1)m}{6} &  \text {if we are in case (1)}, \\ \frac{(\ell +1)m}{2} &  \text {if we are in case (2)}. \end{array}\right. } \end{aligned}$$

If we are in case (1), then $\ell \le \tfrac{1}{6} (\sqrt{24 n+1}-1)$. Similarly, in case (2), $P_{\ell +1}\le \tfrac{1}{6} (\sqrt{24 n+1}-5)$. Using these bounds on $\ell$ and (5.14), we find the lower bound in (5.11), proving the theorem. $\square$

## Proof of Theorem 1.7

In this section we prove Theorem 1.7.

### Proof of Theorem 1.7

By [7, Proposition 2.1] and (1.1), we have

6.1 $$\begin{aligned} U(\zeta ;q)&= \frac{i\sin (\pi z)\eta (\tau )}{q^{\frac{1}{24}}\vartheta (z;\tau )} T(2z;\tau ) + h(\zeta ;q) . \end{aligned}$$

In particular, we have, using (1.5) and the fact that $[\frac{\partial }{\partial z} \vartheta (z;\tau )]_{z=0} = -2\pi \eta ^3(\tau )$,

$$\begin{aligned} U(1;q)&= \frac{T_0(\tau )}{2iq^{\frac{1}{24}}\eta ^2(\tau )}. \end{aligned}$$

Plugging this into (1.9) gives

$$\begin{aligned} U(\zeta ;q)&=\frac{\sin (\pi z) T_0(\tau )}{2\pi iz\eta ^2(\tau )}\exp \left( 2\sum _{k\ge 1} u_k(\tau ) \frac{(2\pi i z)^k}{k!}\right) . \end{aligned}$$

Using this in (6.1), we obtain

$$\begin{aligned} \frac{\sin (\pi z) T_0(\tau )}{2\pi iz\eta ^2(\tau )} \exp \left( 2\sum _{k\ge 1} u_k(\tau ) \frac{(2\pi i z)^k}{k!}\right)&= \frac{i\sin (\pi z)\eta (\tau )}{q^{\frac{1}{24}}\vartheta (z;\tau )} T(2z;\tau ) + h(\zeta ;q). \end{aligned}$$

Equivalently, using (1.3), (1.6), and (1.7), we have

$$\begin{aligned}&\exp \left( 2\sum _{k\ge 1} u_k(\tau ) \frac{(2\pi i z)^k}{k!}\right) \\&= \exp \left( \sum _{k\ge 1} \left( 2G_k(\tau )-2^k g_k(\tau )\right) \frac{(2\pi iz)^k}{k!}\right) + \frac{4\pi z \eta ^3(\tau )}{T_0(\tau )}\exp \left( -\sum _{k\ge 1} h_k(\tau )\frac{(2\pi iz)^k}{k!}\right) .\end{aligned}$$

Using this identity and Lemma 2.3, we obtain

$$\begin{aligned}&\sum _{{k\ge 0}}\sum _{\lambda \vdash k}\prod _{j=1}^k\frac{\left( \frac{2u_j(\tau )}{j!}\right) ^{m_j}}{m_j!}(2\pi iz)^k \\&= \sum _{{k\ge 0}}\sum _{\lambda \vdash k}\prod _{j=1}^k\frac{\left( \frac{2G_j(\tau )-2^j g_j(\tau )}{j!}\right) ^{m_j}}{m_j!} (2\pi iz)^k + \frac{4\pi z\eta ^3(\tau )}{T_0(\tau )} \sum _{{k\ge 0}}\sum _{\lambda \vdash k}\prod _{j=1}^k\frac{\left( \frac{-h_j(\tau )}{j!}\right) ^{m_j}}{m_j!} (2\pi iz)^k. \end{aligned}$$

In the language of partition traces, we have

$$\begin{aligned} \sum _{k\ge 0}\textrm{Tr}_k(\phi ,u;\tau ) (2\pi iz)^k = \sum _{k\ge 0} \textrm{Tr}_k(\phi ,\gamma ;\tau ) (2\pi iz)^k - \frac{2i \eta ^3(\tau )}{T_0(\tau )} \sum _{k\ge 1} \textrm{Tr}_{k-1}(\psi ,h;\tau )(2\pi iz)^k. \end{aligned}$$

Separating the partition $(k^1)$ from the rest, this yields

6.2 $$\begin{aligned} \frac{2u_k(\tau )}{k!}&= - \sum _{\begin{array}{c} \lambda \vdash k\\ \lambda \ne \left( k^1\right) \end{array}} \phi (\lambda ) u_\lambda (\tau )+ \textrm{Tr}_k(\phi ,\gamma ;\tau ) - \frac{2i\eta ^3(\tau )}{T_0(\tau )} \textrm{Tr}_{k-1}(\psi ,h;\tau ). \end{aligned}$$

Using (1.6), we obtain that

6.3 $$\begin{aligned} g_1(\tau )&= -\frac{1}{2\pi i} \frac{\left[ \frac{\partial }{\partial z}T(z;\tau )\right] _{z=0}}{T_0(\tau )}=-\frac{i\eta ^3(\tau )}{T_0(\tau )}. \end{aligned}$$

Plugging this into (6.2) gives (1.10).

Note that the components of $\gamma =\{G_k-2^{k-1}g_k\}_{k\in \mathbb {N}}$ and $h=\{h_k\}_{k\in \mathbb {N}}$ belong to $\widetilde{\mathcal {M}}[g_1,g_2,\ldots ,h_1,$ $h_2,\ldots ]$. Moreover, since $z \mapsto U(e^{2\pi iz};q)$ is even, we have $u_k=0$ for *k* odd by (1.9). In particular $u_1=0$. Using this and (1.10) inductively we have $u_k\in \widetilde{\mathcal {M}}[g_1,g_2,\ldots ,h_1,h_2,\ldots ].$ This space is closed under the action of *D* by Theorems 1.3 and 1.4. Next we show that $\mathcal {U} = \widetilde{\mathcal {M}}[g_1,g_2,\ldots ,h_1,h_2,\ldots ]$. By definition $\mathcal {U}\subseteq \widetilde{\mathcal {M}}[g_1,g_2,\ldots ,h_1,h_2,\ldots ]$. For the other containment, we need to show that $g_k\in \mathcal {U}$ for $k\ge 3$ odd. To see this observe, using (6.3), (2.5), and Theorem 1.3, that

$$\begin{aligned} \frac{1}{g_1}D(g_1)=\frac{1}{\frac{\eta ^3}{T_0}} D\left( \frac{\eta ^3}{T_0}\right) = D\left( \textrm{Log}\left( \frac{\eta ^3}{T_0}\right) \right) = 3D(\textrm{Log}(\eta ))-D(\textrm{Log}(T_0))=-3G_2 + \frac{g_2}{2} -\frac{g_1^2}{2}. \end{aligned}$$

The claim then follows by induction, using Theorem 1.3. This completes the proof. $\square$

## Examples

We have

$$\begin{aligned} g_{1}(\tau )&= -\frac{1}{2} + q + q^{2} - q^{3} - 2 q^{4} - 3 q^{5} + q^{6} + 4 q^{7} + 8 q^{8} + O\!\left( q^{9}\right) \!, \\ g_{2}(\tau )&= q + 2 q^{2} - q^{3} - 5 q^{4} - 11 q^{5} - 2 q^{6} + 12 q^{7} + 38 q^{8} + O\!\left( q^{9}\right) \!, \\ g_{3}(\tau )&= q + 4 q^{2} + q^{3} - 11 q^{4} - 39 q^{5} - 30 q^{6} + 22 q^{7} + 170 q^{8} + O\!\left( q^{9}\right) \!, \\ g_{4}(\tau )&= q + 8 q^{2} + 11 q^{3} - 17 q^{4} - 131 q^{5} - 200 q^{6} - 72 q^{7} + 680 q^{8} + O\!\left( q^{9}\right) \!, \\ g_{5}(\tau )&= q + 16 q^{2} + 49 q^{3} + 13 q^{4} - 399 q^{5} - 1074 q^{6} - 1226 q^{7} + 2078 q^{8} + O\!\left( q^{9}\right) , \\ g_{6}(\tau )&= q + 32 q^{2} + 179 q^{3} + 295 q^{4} - 971 q^{5} - 5072 q^{6} - 10128 q^{7} + 728 q^{8} + O\!\left( q^{9}\right) \end{aligned}$$

and

$$\begin{aligned} h_{1}(\tau )&= -\frac{1}{2} + 2 q + 5 q^{2} + 7 q^{3} + 12 q^{4} + 14 q^{5} + 24 q^{6} + 27 q^{7} + 42 q^{8} + O\!\left( q^{9}\right) \!, \\ h_{2}(\tau )&= 4 q + 17 q^{2} + 37 q^{3} + 83 q^{4} + 140 q^{5} + 273 q^{6} + 425 q^{7} + 736 q^{8} + O\!\left( q^{9}\right) \!, \\ h_{3}(\tau )&= 8 q + 59 q^{2} + 197 q^{3} + 579 q^{4} + 1316 q^{5} + 3019 q^{6} + 5919 q^{7} + 11730 q^{8} + O\!\left( q^{9}\right) \!, \\ h_{4}(\tau )&= 16 q + 209 q^{2} + 1057 q^{3} + 4073 q^{4} + 12032 q^{5} + 32883 q^{6} + 78209 q^{7} + 178426 q^{8} + O\!\left( q^{9}\right) \!, \\ h_{5}(\tau )&= 32 q + 755 q^{2} + 5717 q^{3} + 28887 q^{4} + 108692 q^{5} + 355399 q^{6} + 1007247 q^{7} + 2645982 q^{8} + O\!\left( q^{9}\right) \!, \\ h_{6}(\tau )&= 64 q + 2777 q^{2} + 31177 q^{3} + 206513 q^{4} + 977960 q^{5} + 3828723 q^{6} + 12805745 q^{7} + O\!\left( q^{8}\right) \!. \end{aligned}$$

## Questions for future research

Here we raise some questions for future research.

1. By Proposition 3.2, the function $\widehat{H}$ defined in (3.3) transforms modular on $\Gamma _1(3)$. Note that it could also be viewed as a vector-valued Jacobi form on $\textrm{SL}_2(\mathbb {Z})$. It is thus natural to ask how the remaining components of the associated vector–valued Jacobi form can be described or characterized. Are they related to interesting combinatorial objects?
2. Computational data suggests that the Fourier coefficients of $h_k$ are positive (except for $\operatorname {coeff}_{[q^0]}h_1$ $=-\tfrac{1}{2}$). This raises the question of whether these coefficients possess a combinatorial interpretation. Furthermore, the description of the Fourier coefficients in Theorem 1.6 appears insufficient to establish positivity, since the quantities $b_{n,m}$ are not always positive. Resolving this question might require a more refined analysis or a different approach.
3. In [2, equation (3.7)], analogs of the raising and lowering operators were introduced in the context of the completion of false objects. A natural question is to investigate their actions on $\widehat{T}$ and $\widehat{H}$, as well as their induced actions on $\widehat{g}_k$ and $\widehat{h}_k$.
4. Can one utilize the raising and lowering operators mentioned in the previous question to study the action of heat operator on $\widehat{T}$ and $\widehat{H}$, or equivalently obtain a rank-crank type PDE for $\widehat{T}$ and $\widehat{H}$? This would enable us to understand the spaces generated by $\widehat{g}_k$ and $\widehat{h}_k$, respectively.

## Data Availability

Data sharing is not applicable to this article as no new data were created or analyzed in this study.

## Footnotes

1 The definition of *h* should be compared with the expansion of $\eta$ in the pentagonal number theorem.

2 That is, the addition of a non-trivial function of $\tau$ and $\overline{\tau }$ to produce a quasimodular object.

3 Throughout the paper, we use the notation $\lambda = (1^{m_1}, 2^{m_2}, \dots , n^{m_n}) \vdash n$ to denote a partition of *n*, where $m_j$ is the multiplicity of *j*.

4 A sequence of integers, such as (2, 2), may give rise to multiple unimodal sequences; we use an underline on *c* to denote that it is the peak of the unimodal sequence.

5 This lemma was also used in [4].

6 For detailed study of Jacobi forms we refer the readers to Eichler–Zagier [5].
